# Supplementary material for: Versatile Encapsulation and Synthesis of Potent Liposomes by Thermal Equilibration
Source: Membranes (Basel). 2022 Mar 11;12(3):319. doi: 10.3390/membranes12030319 (PMC8954264; doi:10.3390/membranes12030319)
Supplement: Supplementary file 1 [file membranes-12-00319-s001.zip › membranes-1591874-supplementary.pdf]

Supplementary Information

Simulation System

Simulations were performed on a lipid membrane system consisting of DSPC and cholesterol molecules in a 2:1 molar ratio respectively. Since all-atom MD simulations are computationally demanding, a section of the liposomal membrane was used to represent the thermodynamic changes observed at a local point within a liposome that allow the passive diffusion of small molecules like drugs. Charmm GUI[1,2] *Membrane Builder* was used to build the membrane system. The lipid bilayer was built in a rectangular box containing a total of 256 lipid molecules (128 on each layer of the bilayer known as a leaflet) and 128 cholesterol molecules (64 on each leaflet). Additionally, 30 water molecules per lipid molecule were present to ensure hydration of the lipid head. The effect of different temperatures on different lipid molecules (like DLPC, DMPC, and DPPC) has been previously studied by Zhuang *et. al*[3], using a bilayer of 72 lipid molecules (36 on each leaflet). The number of lipid molecules was increased by ~3.5 times for our simulations to build a bigger model representing the liposome. Furthermore, a bilayer system containing 256 molecules of DSPC has been previously used by Magarkar *et al*[4] who studied a molecular dynamics model of the liposome surface in the bloodstream. The L_x and L_y components of the bilayer are 10.5 nm with an L_z component of 4.25 nm. The total number of molecules in the system is given in Table 1.

Table S1. Molecular Dynamic Simulation system information.

DSPC	Cholesterol	Water	Simulation temperatures (C)	Simulation time (ns)
256	128	5534	37, 55, 65	100

Molecular Dynamics simulation setup:

The simulation used a standard TIP3 water model[5,6] and CHARMM36[7] lipid parameter set combined with the CHARMM parameters for the general force field of small molecules (CGenff)[8]. A neutralizing salt (NaCl) concentration of 0.05 mol/L was added to the system[9]. Periodic boundary conditions were applied in all three dimensions. Van der Waals interactions were switched off between 10 and 12 Å by a force-based switching function[10]. In each simulation, Particle Mesh Edwald (PME)[11] was used for electrostatic interactions. NAMD[12,13] (Nanoscale Molecular Dynamics) was used to thermally equilibrate the bilayer system for 4 ns. RATTLE algorithm[14] was used to constrain all bond lengths involving hydrogen atoms.

All thermally equilibrated systems were used for simulation runs at different temperatures (Table 1). The simulation time step was 1fs and the data was collected every 20 ps. A simulation for each temperature was run for 100 ns. NPT ensemble was used for simulations with the pressure at 1 bar maintained by Nosè-Hoover Langevin[15] piston method. A constant temperature in each simulation was maintained by Langevin thermostat. The trajectories were visualized to create snapshots of the bilayer using Visual Molecular Dynamics (VMD).

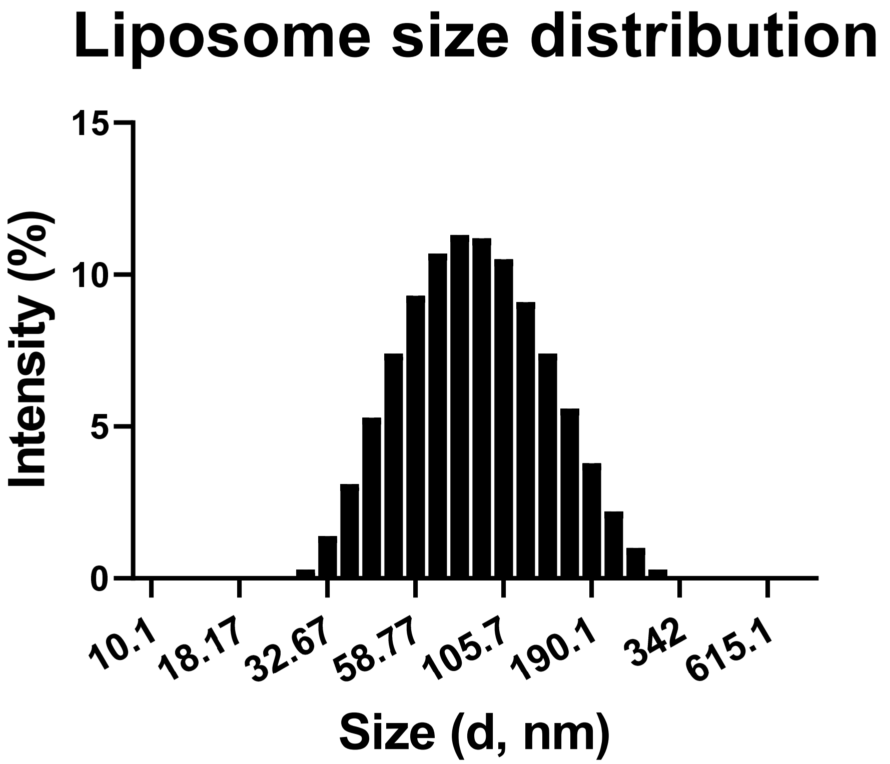

Figure S1. The size distribution of liposomes. The average diameter of the liposome population was measured using dynamic light scattering (DLS). The average size was 79.56 (± 4.81, PDI 0.183) nm.

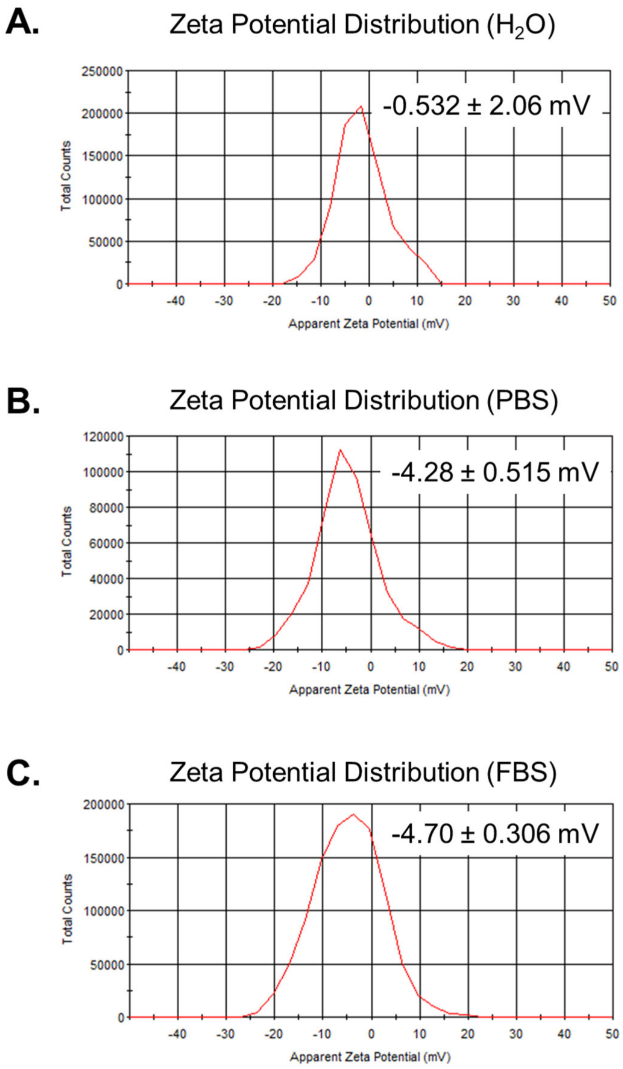

Figure S2. The zeta potential of liposomes in H_2O, PBS, FBS was -0.532 ± 2.06mV, -4.28 ± 0.515 mV, and -4.70 ± 0.306 mV respectively, measured using Malvern Zetasizer ZSP dynamic light scattering system.

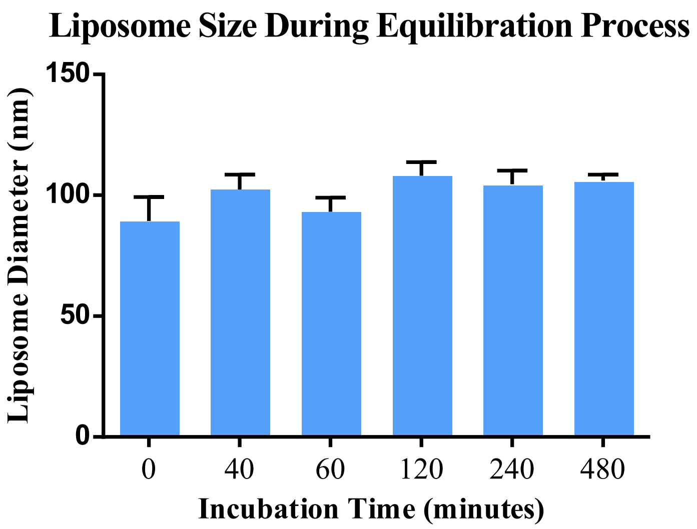

Figure S3. Diameter of Liposomal DXR following ET. The diameter of liposomes with increasing equilibration durations at 55 ^{O}C was analyzed via DLS. The distribution of liposome diameter does not display a significant change over 480 minutes. This demonstrates the stability of the liposomes and is also used to evaluate the maximum equilibration concentration.

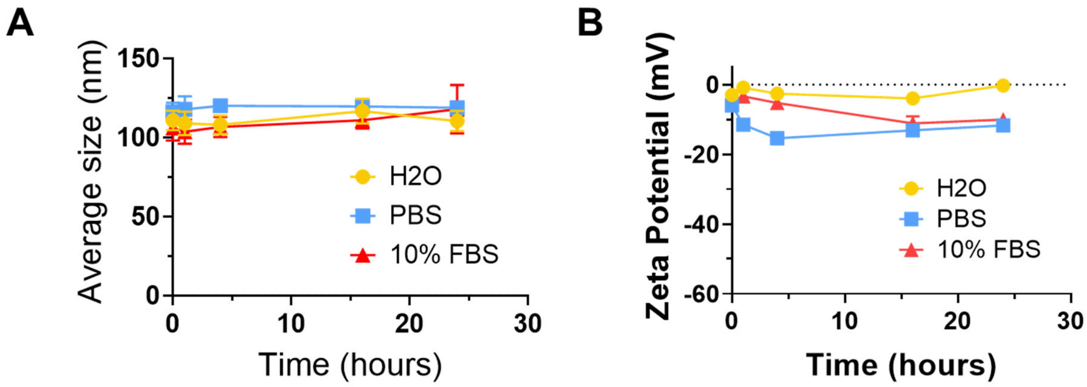

Figure S4. The stability of liposomes in serum was analyzed and compared with their stability in H_2O and PBS. Lipid nanoparticles were dispersed in either H_2O, PBS, or 10% FBS in PBS and incubated at 37 ºC, and the particle size and zeta potential were recorded at each time point (0, 1, 4, 16, and 24 hours). **A.** The particle size change was negligible when the liposomes were incubated in H_2O or PBS for up to 24 hours with a change of 0.003 % and 2.11% respectively. When the particles were mixed with 10% FBS, the particle size increased from 105.9 ± 10.2 nm to 117.8 ± 15.3 nm likely due to protein absorption on the particle surface. **B.** The liposome zeta potential did not change when the liposomes were incubated in H_2O, but it reduced from -5.95 ± 0.478 to -11.7 ± 0.30 and from -4.70 ± 0.306 to -10.05 ± 0.404 when the liposomes were incubated in PBS or 10% FBS respectively.

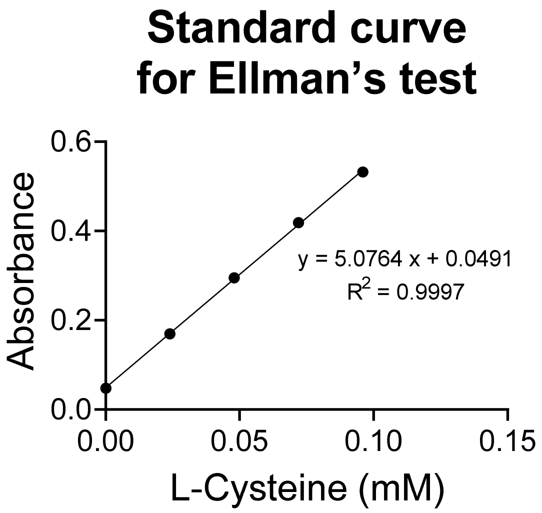

	Average	std
Absorbance	0.092	0.011
DSPE-PEG-MAL (mM)	0.059	0.003
DSPE-PEG-MAL/DSPC ratio	0.009	0.001

Figure S5. Quantification of Maleimide:Lipid ratio was evaluated by Ellman's test. The result showed that the maleimide to DSPC lipid ratio is 0.009 which is close to its initial value of 0.01. Since the simulations were conducted on a section of the membrane with 256 lipids and 128 cholesterol, about 2-3 DSPE-PEG-MAL should be comprised within this region.

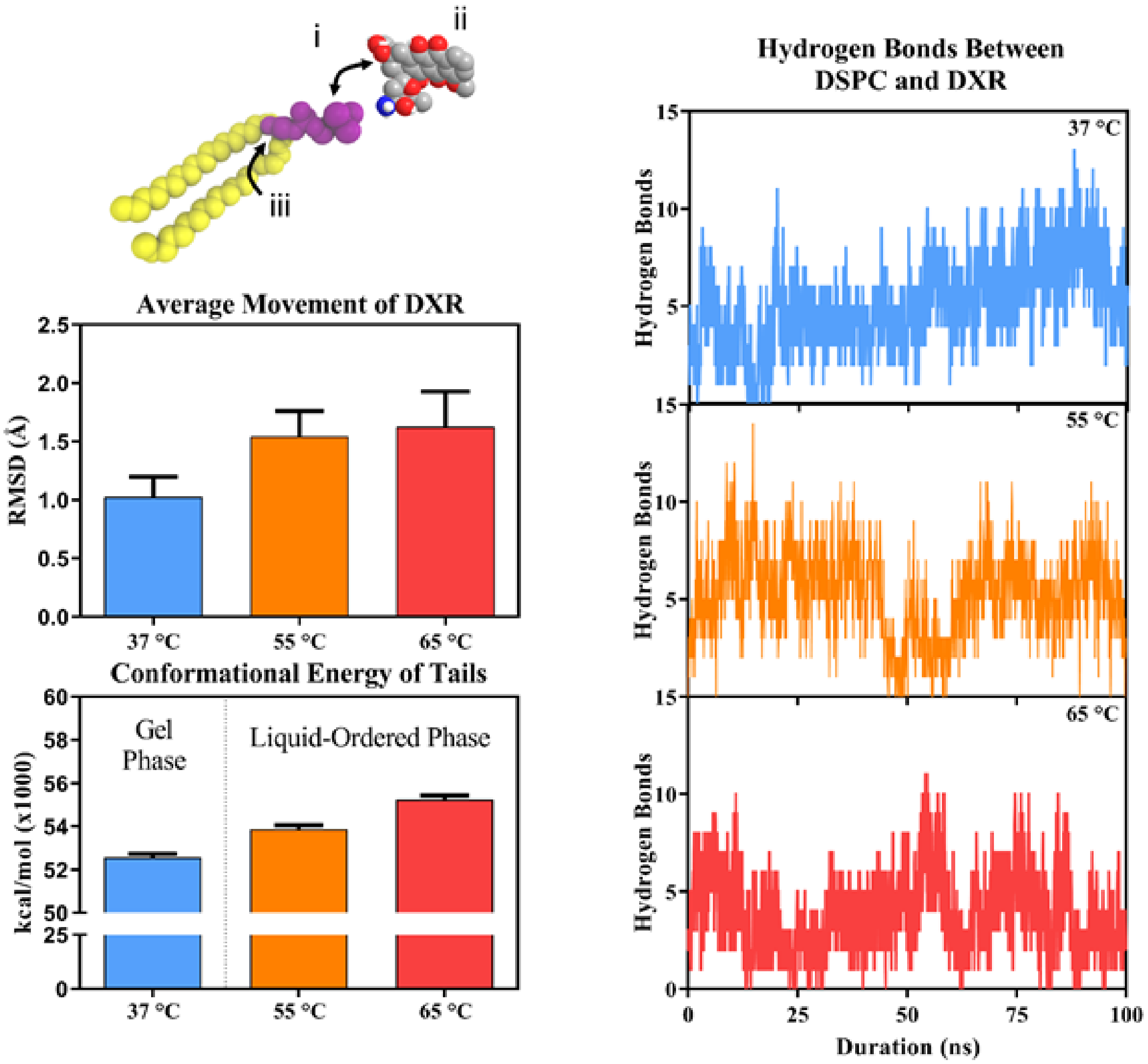

Figure S6. Effects of increased temperature on DXR equilibration. (A) The stability of the DXR-lipid interactions is dictated by several factors including (i) hydrogen bonds, (ii) Brownian motion and diffusion of the DXR, and (iii) conformational energy of the tails which allows for diffusion through the membrane. (B) The DXR can be stabilized both inside and outside the liposome through hydrogen bonding between the oxygen and nitrogen atoms on the DXR and phosphates on the DSPC. (C) This stabilization, along with VdW and electrostatic interactions can have a profound effect on the net movement of the DXR through the liposome. (C) While temperature clearly has an effect on the fluidity of the membrane, it also plays a role in decreasing the liposome stability. Both the transition from 37 OC to 55 OC and from 55 OC to 65 OC result in an increase in conformational energy of 2.5%.

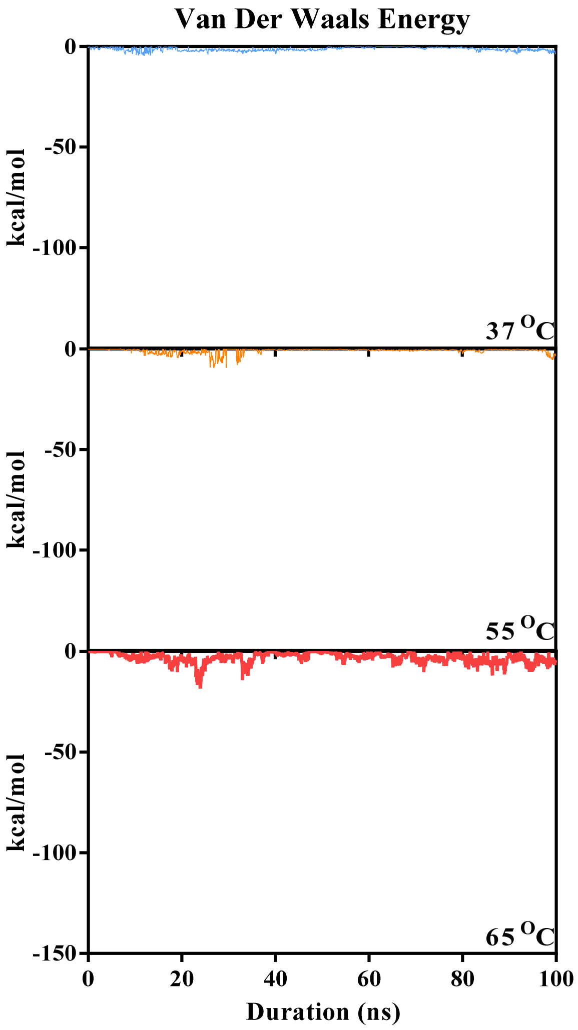

Figure S7. Long-range VdW interactions between DXR and Cholesterol. VdW forces between cholesterol and DXR are minimal. At 65 ^{O}C, lipid tails are characterized by higher motion exposing cholesterol molecules to DXR accounting for slight interactions between DXR and cholesterol as seen in the figure above. Below T_m, thermal movement of tails is limited and DXR is bound to lipid heads with no interactions with cholesterol. At T_m, motions of tails are fluid but ordered which does not favor interactions between DXR and cholesterol. Cholesterol provides structural rigidity to liposomes. A higher interaction between DXR and cholesterol may affect the structural function of cholesterol and aid in the rapid breakdown of liposomes.

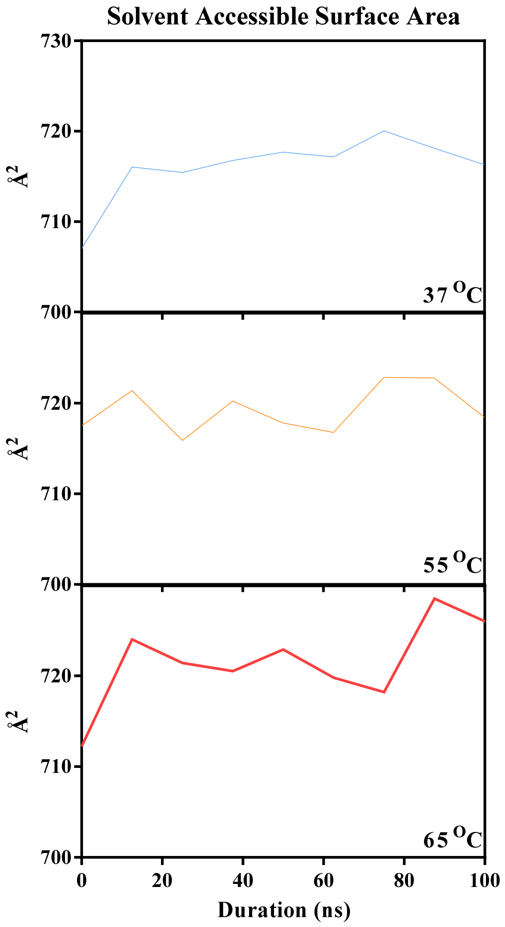

Figure S8. Solvent Accessible Surface Area (SASA) of lipids at increasing temperatures analyzed at intervals of 10 ns. With increasing temperatures, SASA values showed a steady rise. A higher area of lipids is exposed to water due to fluctuations of the tails. The increased water exposed area with higher temperatures is another factor that contributes to the destabilization of the membrane. As the temperatures increased, SASA also increased and could be a reason for the increased DXR movement into the lipid membrane during the simulation as well as destabilization of the membrane at higher temperatures

Supplementary Citations

1. Jo, S., Kim, T., Iyer, V. G. & Im, W. CHARMM-GUI: a web-based graphical user interface for CHARMM. *J. Comput. Chem.* **29,** 1859–1865 (2008).
2. Jo, S., Lim, J. B., Klauda, J. B. & Im, W. CHARMM-GUI Membrane Builder for Mixed Bilayers and Its Application to Yeast Membranes. *Biophys. J.* **97,** 50–58 (2009).
3. Zhuang, X., Makover, J. R., Im, W. & Klauda, J. B. A systematic molecular dynamics simulation study of temperature dependent bilayer structural properties. *Biochim. Biophys. Acta BBA - Biomembr.* **1838,** 2520–2529 (2014).
4. Magarkar, A., Karakas, E., Stepniewski, M., Róg, T. & Bunker, A. Molecular Dynamics Simulation of PEGylated Bilayer Interacting with Salt Ions: A Model of the Liposome Surface in the Bloodstream. *J. Phys. Chem. B* **116,** 4212–4219 (2012).
5. Jorgensen, W. L., Chandrasekhar, J., Madura, J. D., Impey, R. W. & Klein, M. L. Comparison of simple potential functions for simulating liquid water. *J. Chem. Phys.* **79,** 926–935 (1983).
6. Macwan, I. *et al.* Interactions between avidin and graphene for development of a biosensing platform. *Biosens. Bioelectron.* **89,** 326–333 (2017).
7. Klauda, J. B. *et al.* Update of the CHARMM All-Atom Additive Force Field for Lipids: Validation on Six Lipid Types. *J. Phys. Chem. B* **114,** 7830–7843 (2010).
8. MacKerell, A. D. *et al.* All-atom empirical potential for molecular modeling and dynamics studies of proteins. *J. Phys. Chem. B* **102,** 3586–3616 (1998).
9. Xing, J. *et al.* Increasing vaccine production using pulsed ultrasound waves. *PloS One* **12,** e0187048 (2017).

10. Steinbach, P. J. & Brooks, B. R. New spherical-cutoff methods for long-range forces in macromolecular simulation. *J. Comput. Chem.* **15,** 667–683 (1994).
11. Darden, T., York, D. & Pedersen, L. Particle mesh Ewald: An N·log(N) method for Ewald sums in large systems. *J. Chem. Phys.* **98,** 10089–10092 (1993).
12. Phillips, J. C. *et al.* Scalable molecular dynamics with NAMD. *J. Comput. Chem.* **26,** 1781–1802 (2005).
13. Ray, A., Macwan, I., Singh, S., Silwal, S. & Patra, P. A Computational Approach for Understanding the Interactions between Graphene Oxide and Nucleoside Diphosphate Kinase with Implications for Heart Failure. *Nanomaterials* **8,** 57 (2018).
14. Andersen, H. C. Rattle: A "velocity" version of the shake algorithm for molecular dynamics calculations. *J. Comput. Phys.* **52,** 24–34 (1983).
15. Feller, S. E., Zhang, Y., Pastor, R. W. & Brooks, B. R. Constant pressure molecular dynamics simulation: The Langevin piston method. *J. Chem. Phys.* **103,** 4613–4621 (1995).
